# Health Impact of Outdoor Air Pollution in China: Current Knowledge and Future Research Needs

**DOI:** 10.1289/ehp.12737

**Published:** 2009-05

**Authors:** Haidong Kan, Bingheng Chen, Chuanjie Hong

**Affiliations:** Fudan University, Shanghai, China, E-mail: haidongkan@gmail.com

Outdoor air pollution is one of China’s most serious environmental problems. Coal is still the major source of energy, constituting about 75% of all energy sources. Consequently, air pollution in China predominantly consists of coal smoke, with suspended particulate matter (PM) and sulfur dioxide (SO_2_) as the principal air pollutants. In large cities, however, with the rapid increase in the number of motor vehicles, air pollution has gradually changed from the conventional coal combustion type to the mixed coal combustion/motor vehicle emission type. Currently, inhalable particles (PM <10 μm in aerodynamic diameter; PM_10_), SO_2_, and nitrogen dioxide (NO_2_) are the criteria pollutants of concern in China. Generally, PM levels in cities in the north are higher than those in the south, whereas SO_2_ and NO_2_ levels do not differ much. In 2004, the annual average PM_10_ concentrations for major Chinese cities were 102 μg/m^3^ in southern cities, 140 μg/m^3^ in northern cities, and 121 μg/m^3^ in cities nationwide. The annual average concentrations of SO_2_ and NO_2_ nationwide were 66 μg/m^3^ and 38 μg/m^3^, respectively (China State Environmental Protection Agency 2005).

Although its ambient air quality has improved substantially, China is still facing the worst air pollution problem in the world. Outdoor air pollution has become a major concern for public health. The World Bank (2007) estimated that the total health cost associated with outdoor air pollution in urban areas of China in 2003 was between 157 and 520 billion Chinese yuan, accounting for 1.2–3.3% of China’s gross domestic product. Health end points studied in China in association with air pollution include all-cause mortality, mortality and morbidity due to cardiopulmonary disease, and numbers of outpatient and emergency department visits (Chen et al. 2004). Changes in respiratory and other clinical symptoms, lung function, and immune function are also studied.

Dozens of time-series studies have been conducted in large Chinese cities, including Beijing, Shanghai, Chongqing, Shenyang, Wuhan, and Taiyuan, to assess the association of short-term exposure to air pollution with mortality or morbidity (Chen et al. 2004). Mortality or morbidity risk estimates per unit increase in air pollution level among Chinese populations are generally similar in magnitude to risks estimated in other parts of the world. A recent multicity time-series analysis in Hong Kong, Shanghai, and Wuhan provided further evidence of short-term risks (Wong et al. 2008), with significant health effects detected at air pollution levels below minimum air quality standards in China. Currently, a new national-level air pollution time-series study, the China Air Pollution and Health Effects Study (CAPES), is under way. In addition, several ongoing panel studies are examining associations between air pollution and subclinical health outcomes before, during, and after the 2008 Summer Olympic Games in Beijing. These panel studies should provide a unique opportunity to assess the public health benefits of air pollution reduction in a city where air pollution levels have been high.

Relatively few studies have examined long-term effects of air pollution in China. Several prospective cohort studies in North America and Europe have estimated effects of long-term exposure to air pollution on mortality (Pope and Dockery 2006), but it is not clear whether the findings from developed countries apply to China, given differences in the levels and characteristics of air pollution, and in sociodemographic characteristics. So far, there has been no cohort study of air pollution in China, but results of cross-sectional analyses in Beijing, Shenyang, and Benxi have suggested that long-term exposure to air pollution is associated with increased mortality (Chen et al. 2004). However, results of these analyses are difficult to interpret because of the lack of information on potential confounders.

In short, there is sufficient evidence that exposure to outdoor air pollution is a health hazard in China. The importance of these increased health risks is greater than in developed countries because air pollution in China is at much higher levels and because the Chinese population accounts for more than one-fourth of the world’s total population. Most of the Chinese studies discussed above were ecologic in nature, thus limiting their power for causal inference. Future research will need to clarify the lifetime course of air pollution effects with full control of potential confounders (e.g., prospective cohort studies), examine the relevance of cumulative exposures, disentangle effects of multiple pollutants, investigate gene–environment inter actions and other factors that may modify air pollution health effects, and identify pathophysiologic links between air pollution and health hazards for the Chinese population. Finally, pollution needs to be reduced and air quality and health indicators need to be monitored; this will enable the people and relevant authorities to be aware of the trends and consequences of air pollution, so they can determine how to ameliorate the situation.

## Figures and Tables

**Figure:**
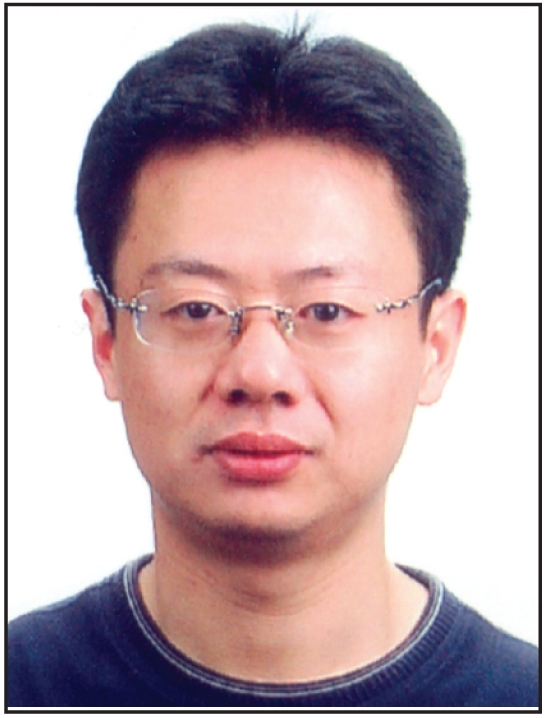
Haidong Kan

**Figure:**
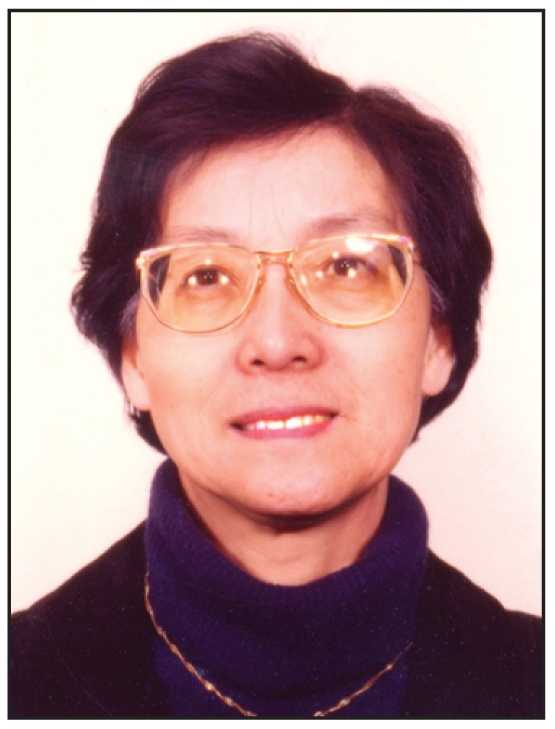
Bingheng Chen

**Figure:**
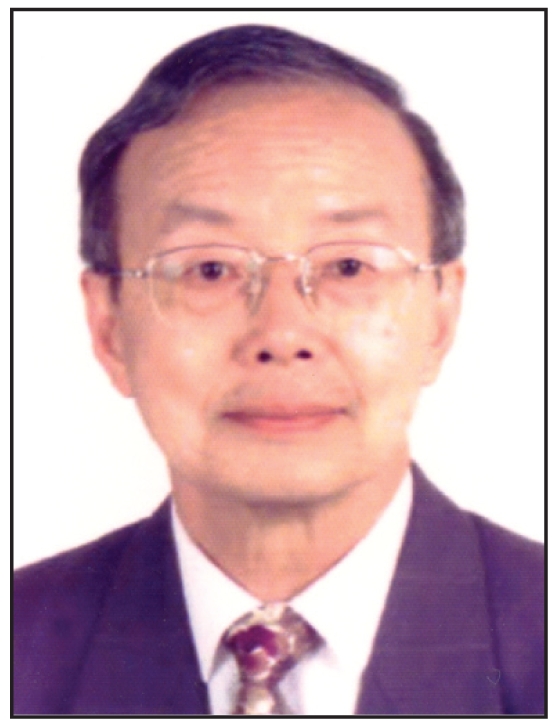
Chuanjie Hong
